# Predicting Sleep Quality Based on Metabolic, Body Composition, and Physical Fitness Variables in Aged People: Exploratory Analysis with a Conventional Machine Learning Model

**DOI:** 10.3390/jfmk10030337

**Published:** 2025-09-02

**Authors:** Pedro Forte, Samuel G. Encarnação, José E. Teixeira, Luís Branquinho, Tiago M. Barbosa, António M. Monteiro, Daniel Pecos-Martín

**Affiliations:** 1 Physiotherapy and Pain Group, Department of Physical Therapy, University of Alcala, 28801 Madrid, Spain; daniel.pecos@uah.es; 2 Department of Sports, Higher Institute of Educational Sciences of the Douro, 4560-708 Penafiel, Portugal; samuel01.encarnacao@gmail.com; 3 Research Centre for Active Living and Wellbeing (LiveWell), Instituto Politécnico de Bragança, 5300-253 Bragança, Portugal; jose.eduardo@ipg.pt (J.E.T.); barbosa@ipb.pt (T.M.B.); mmonteiro@ipb.pt (A.M.M.); 4 Department of Sports Sciences, Instituto Politécnico de Bragança, 5300-253 Bragança, Portugal; 5 Department of Physical Activity and Sport Sciences, Universidad Autónoma de Madrid (UAM), 28049 Madrid, Spain; 6 Department of Sports Sciences, Polytechnic of Guarda, 6300-559 Guarda, Portugal; 7 Department of Sports Sciences, Polytechnic of Cávado and Ave., 4800-058 Guimarães, Portugal; 8 SPRINT—Sport Physical Activity and Health Research & Inovation Center, 6300-559 Guarda, Portugal; 9 Biosciences Higher School of Elvas, Polytechnic Institute of Portalegre, 7350-000 Portalegre, Portugal; luisbranquinho@ipportalegre.pt; 10 Life Quality Research Center (LQRC-CIEQV), 2001-904 Santarém, Portugal

**Keywords:** age, sleep quality, physical fitness, body composition

## Abstract

**Background**: Sleep plays a crucial role in the health of older adults, and its quality is influenced by multiple physiological and functional factors. However, the relationship between sleep quality and physical fitness, body composition, and metabolic markers remains unclear. This exploratory study aimed to investigate the associations between sleep quality and physical, metabolic, and body composition variables in older adults, and to evaluate the preliminary performance of a logistic regression model in classifying sleep quality. **Methods**: A total of 32 subjects participated in this study, with a mean age of 69. The resting arterial pressure (systolic and diastolic), resting heart rate, anthropometrics (high waist girth), body composition (by bioimpedance), and physical fitness (Functional Fitness Test) and sleep quality (Pitsburg sleep-quality index) were evaluated. Group comparisons, associative analysis and logistic regression with 5-fold stratified cross-validation was used to classify sleep quality based on selected non-sleep-related predictors. **Results**: Individuals with good sleep quality showed significantly better back stretch (t = 2.592; *p* = 0.015; η^2^ = 0.239), lower limb strength (5TSTS; t = 2.564; *p* = 0.016; η^2^ = 0.476), and longer total sleep time (t = 6.882; *p* < 0.001; η^2^ = 0.675). Exploratory correlations showed that poor sleep quality was moderately associated with reduced lower-limb strength and mobility. The logistic regression model including 5TSTS and TUG achieved a mean accuracy of 0.76 ± 0.15, precision of 0.79 ± 0.18, recall of 0.83 ± 0.21, and AUC of 0.74 ± 0.16 across cross-validation folds. **Conclusions**: These preliminary findings suggest that physical fitness and clinical variables significantly influence sleep quality in older adults. Sleep-quality-dependent patterns suggest that interventions to improve lower limb strength may promote better sleep outcomes.

## 1. Introduction

Aging leads to significant changes in body composition and functional fitness [1]. These changes, notably the reduction in muscle mass and increase in adiposity, are associated with a higher risk of conditions like metabolic syndrome [1], and may lead to sarcopenia, contributing to frailty and loss of independence in older adults [2]. Physiologically, aging also affects hemodynamic parameters, including pulse pressure, arterial stiffness, and wave reflections, particularly in large arteries [3], leading to increased pulse pressure, especially in individuals over 60 years old [4].

Age-related alterations in body composition, functional fitness, resting heart rate, and arterial blood pressure are intricately linked to sleep quality [5]. In particular, obesity in older adults has been associated with reduced sleep duration, suggesting a potential interaction between metabolic and sleep-related factors [6]. Healthy behaviors may moderate this relationship, with evidence indicating that positive lifestyle factors contribute to improved sleep outcomes [7]. For example, Wu et al. [8] conducted a meta-analysis involving 197,906 participants, showing that obesity significantly shortens sleep duration. Moreover, overweight and obese individuals typically exhibit a pro-inflammatory profile with elevated levels of cytokines such as tumor necrosis factor α (TNF-α), interleukin 6 (IL-6), and C-reactive protein (CRP) [9,10], which disrupts sleep regulation by impairing hypothalamic control of non-rapid eye movement (Non-REM) sleep [11,12].

Physical exercise and improved fitness adaptations have also been shown to positively influence sleep regulation [13,14]. Several physiological mechanisms underlie this effect, including reductions in depression and anxiety [15], improved thermoregulation following physical exertion [16,17], enhanced muscle relaxation [18], and hormonal regulation involving melatonin, cortisol, growth hormone, adenosine, ghrelin, leptin orexin, prolactin, and serotonin [19,20]. These pathways support the hypothesis that maintaining or improving physical fitness may contribute to better sleep quality in older populations.

To assess physical fitness and function in elderly individuals, various tests have been employed, including the Eurofit battery [21], the Wii Fit Balance Board [22], and the Physical Activity Scale for Elderly (PASE) [23]. More commonly used assessments include the Timed Up and Go Test (TUG) and Tinetti Gait and Balance Test, which evaluate balance and fall risk [24,25], as well as handgrip strength, an indicator of motor function and overall health [26]. Among the most used protocols, the Fullerton Functional Fitness Test (FFFT) [27], developed by Rikli and Jones, comprehensively assesses strength, flexibility, coordination, and aerobic fitness [28]. This battery includes components such as strength, balance, coordination, flexibility, and aerobic fitness [29,30]. Many studies examining physical fitness in older adults also include body composition metrics to gain a more complete health profile [31,32,33].

Despite the known links between sleep quality, physical fitness, and metabolic health, the evidence base in older populations remains limited [34]. However, there are associations between metabolic rates and sleep quality, but research in aged adults is rare [35,36,37,38,39]. The only research found was the Schilling et al. [40] study. Additionally, Kohanmoo et al. [41] and Tan et al. [42] reported an inverse relationship between fat mass and sleep quality or duration. However, most prior studies examined these domains in isolation, without integrating physical, metabolic, and sleep-related variables into a unified predictive model.

Poor sleep quality in older adults (short duration and frequent awakenings) impairs recovery, increases cortisol and anxiety levels, and perpetuates fatigue and reduced quality of life [34]. It is also associated with worse mental health and diminished performance in daily activities [43]. Considering these challenges, predictive models that integrate multiple health domains may help identify older individuals at risk of poor sleep quality. Emerging tools such as machine learning (ML) have shown promise in modulating complex health outcomes. By processing multidimensional data, ML can uncover non-linear relationships and offer preliminary classification capabilities, even in exploratory settings. However, few studies have applied ML to predict sleep quality using physical fitness and body composition variables in older adults. Therefore, the present study aimed to compare clinical and functional fitness variables by sleep quality, and to assess the predictive value of these variables using machine learning algorithms. It was hypothesized that body composition, functional fitness, and metabolic variables would significantly predict sleep quality.

## 2. Materials and Methods

### 2.1. Study Design

This study followed a cross-sectional observational design, to explore associations between body composition, functional fitness, and sleep quality in older adults. A total of 32 community-dwelling individuals aged 60 years or older were assessed in a controlled, single-session setting. Participants were recruited from a local health and exercise program, using a convenient sampling approach. Therefore, all results should be interpreted as exploratory and hypothesis-generating, laying the groundwork for future studies with larger and more representative samples. Sleep quality was evaluated using the Pittsburgh Sleep Quality Index (PSQI), a validated subjective tool, while body composition was estimated using a bioimpedance scale (Tanita BC-601). The functional fitness test was used, and other parameters, including the sit-to-stand test, handgrip strength, and walking speed, were evaluated. All procedures were conducted by trained researchers following standardized protocols to ensure consistency across measurements. This study adhered to key elements of the STROBE checklist for cross-sectional studies, including clear reporting of variables, participant characteristics, statistical methods, and limitations.

### 2.2. Sample

Thirty-two subjects participated in this study; twenty-six were females, and six were males. The sample mean age was 69 years. The convenience sample was recruited in the Bragança Municipality. All the participants were aged community people. All the procedures were in agreement with Helsinki’s declaration. The research project received approval by the Ethical Committee of the Instituto Politécnico de Bragança (number: 2576). The participants were instructed to maintain normal daily activities to prevent physical inactivity. The participants were asked to complete a sample characterization questionnaire during the first visit. The inclusion criteria were: (i) being aged 60 years or older, (ii) maintaining functional independence in daily activities; (iii) not having severe chronic diseases or taking sleep-related medication; (iv) not having any significant cardiovascular, musculoskeletal, metabolic, or joint conditions that could interfere with the assessments; (v) not having developed any new illness or begun any new medication during the study period that could affect sleep or physical function; (vi) being a non-smoker; (vii) not having undertaken long-distance travel during the study period that could cause jet lag and affect sleep quality. Given the small sample size, the results are exploratory in nature and not generalizable.

### 2.3. Procedures

#### 2.3.1. Anthropometrics, Body Composition, and Metabolic Variables

Anthropometrics was evaluated by stature and body mass. Additionally, the body composition was estimated with a digital bioimpedance scale (Tanita BC-601, Arlington Heights, IL, USA), which is validated and used for research [44]; however, this equipment does not provide the estimation algorithms. The computed variables of body composition were lean mass, percentage of fat mass, bone mineral density, visceral fat, total body mass, muscle mass, fat mass, and bone mineral density. The Tanita BC-601 device uses proprietary algorithms to estimate body composition and does not provide raw impedance values. These estimates may not be fully validated for all populations, especially older adults with atypical body composition. Therefore, results should be interpreted with caution. The participants made the evaluations wearing light clothing and without shoes and socks during the morning and before breakfast. It is important to note that the use of BIA in elderly populations presents inherent limitations, as age-related alterations in fluid balance and body tissue conductivity may compromise measurement accuracy. The stature was evaluated standing with the head in the Frankfurt plane. Waist and hip circumferences were also evaluated. The cardiovascular measures variables were the arterial pressure (systolic and diastolic) and resting heart rate measured with an OMRON (M2 HEM-7143-E, Omron, Kyoto, Japan), which is also validated to be used in research [44]. The metabolic rate was estimated by bioimpedance with the Tanita.

#### 2.3.2. Arterial Blood Pressure, Resting Heart Rate and Sleep Quality

Arterial systolic blood pressure (SBP), diastolic blood pressure (DBP), and resting heart rate (RHR) were measured following the 2018 European Society of Cardiology and the European Society of Hypertension (ESC/ESH) Guidelines for the management of arterial hypertension [45]. Two measurements were performed, and the average between metrics was calculated.

Sleep quality was verified through the use of the Pitsburg sleep-quality index (PSQI), a 19-item questionnaire [46], and validated for the Portuguese population [47], used in this research. The PSQI items are subdivided into the following components: (1) subjective sleep quality, (2) sleep latency, (3) sleep duration, (4) habitual sleep efficiency, (5) sleep disturbances, (6) use of sleeping medication, and (7) daytime dysfunction. Each component is scored from 0 to 3, with higher scores indicating poorer sleep quality. A global score greater than 5 indicates poor sleep quality [47].

#### 2.3.3. Handgrip Strength

Handgrip strength was assessed using a digital palmar dynamometer (CAMRY^®^, Lisbon, Portugal), with the maximum kilograms-force (kgf) achieved using a palm grip as the measurement. The participants stood with their arms away from their bodies and, upon the researcher’s signal, exerted maximum palm grip force on the dynamometer for four seconds [29]. Each participant was given two attempts, and the evaluator noted the highest recorded result.

#### 2.3.4. Functional Fitness

The Functional Fitness Test by Rikli and Jones was used to assess the main physical parameters associated with functional mobility [28]. The battery was composed of the 2 min Step Test and the Seat To Stand, where each participant was positioned standing up in front of a 43 cm highchair. In the arm curl test, the participant was positioned in a chair 43 cm high, holding a 2 kg dumbbell. The Time-Up-and-Go Test was conducted with the participant seated in a chair 43 cm high, facing a cone at 2.44 m, and the time was recorded in seconds after two trials. Finally, the Sit and Reach and in the Back Scratch tests were applied.

#### 2.3.5. Relative Lower Limb Muscle Power

The lower limb muscle power was measured through the five-time sit-to-stand (5TSTS) test. The test was performed in a standardized chair of 0.49 m in height. The evaluator encouraged the participants throughout the test to ensure they always perform the maximum movement speed and preserve the technique. Two attempts were performed with an interval of 60 s, and the shortest time was noted. Shorter completion time indicates greater lower-limb muscle power [48].

### 2.4. Statistical Analysis

The Kolmogorov–Smirnov test, kurtosis (<±3), and Skewness (−2 to +2 criteria) values allowed us to assess the normality of the distribution, and Levene’s test assessed the homogeneity. Thus, the *t*-test allowed the comparison of variables’ sleep quality, and Pearson’s correlation test allowed the association between the variables. The test was carried out at a significance level of 5%. The effect size (eta square − η^2^) was computed and interpreted as without effect (if 0 < η^2^ ≤ 0.04), minimum (if 0.04 < η^2^ ≤ 0.25), moderate (if 0.25 < η^2^ ≤ 0.64), and strong (if η^2^ > 0.64). The logistic regression Machine Learning algorithm was developed using two lower-body functional performance variables: the Five-Time Sit-to-Stand Test (5TSTS1) and the Timed Up and Go Test (TUG), with only two predictors to avoid overfitting (hence, the sample size still limits the power and generalizability of our results). The model was evaluated using 5-fold stratified cross-validation, with standardization of input variables applied within each fold. Performance metrics (accuracy, precision, recall, and ROC AUC) were computed for each fold and reported as mean ± standard deviation. Due to the limited sample size, default hyperparameters were used, and no optimization procedures (e.g., grid search) were performed to avoid overfitting. The 5-fold stratified cross-validation procedure was used to assess model performance. Evaluation metrics included accuracy, precision, recall, and the area under the receiver operating characteristic curve (ROC AUC), reported as mean ± standard deviation. The machine learning procedure was conducted in agreement with the literature for analysis over 20 participants [46]. All statistical analyses were performed using JASP version 0.18 (JASP Team, Amsterdam, The Netherlands), and the machine learning models were developed using Python 3.11 with the Scikit-learn library

## 3. Results

The analyses conducted in this study were structured to address two main objectives: (1) to compare clinical and functional variables according to sleep quality classification; and (2) to evaluate whether these variables could predict sleep quality using machine learning models. Descriptive statistics and group comparisons (*t*-tests and effect sizes) were used to explore differences by sleep quality status. Correlational analyses were performed to examine the relationships between physical fitness, body composition, and sleep quality components. Finally, supervised machine learning models were applied to assess the predictive value of selected variables for classifying sleep quality, providing a preliminary test of their potential in data-driven risk identification.

### 3.1. Descriptives

The study evaluated various health and fitness parameters among 32 individuals, distinguishing between those with good (15) and poor (17) sleep quality. The mean age was 69.28 years. Those with poor sleep demonstrated slightly better performance in the 5-time sit-to-stand test (6.75 s) than those with good sleep quality (7.69 s). These descriptive results provide the context for the subsequent analyses, beginning with comparisons between good and poor sleep quality.

### 3.2. Sleep Quality Comparisons

Descriptives (means and standard deviations) for good and poor sleep quality are presented in Table 1. Poor sleep quality was associated with lower total sleep (3.35 h) compared to good sleep quality (6.13 h). Individuals with poor sleep also had higher mean ages (73.24 years) and visceral fat (8.29) compared to those with good sleep (69.47 years and 7.47 visceral fat). Despite these differences, both groups had similar heart rates (72.38 vs. 71.82 bpm) and total fat percentages (29.93% vs. 32.13%). Comparing the variables between poor and good sleep quality, it is possible to find that the TUG (t = 2.564; *p* = 0.016; η^2^ = 0.476), total fat (kg) (t = 2.592; *p* = 0.015; η^2^ = 0.239), and total sleep (t = 6.882; *p* < 0.001; η^2^ = 0.675) time significantly differed between groups, where the persons with good sleep quality presented higher scores.

Beyond group differences, we then examined associations between the studied variables to better understand the interrelationships underlying sleep quality.

### 3.3. Associative Analysis and Machine Learning

Based on the Pearsons correlation test, the total sleep scores presented significant associations with 5TSTS (r = 0.442; *p* = 0.011), TUG (r = 0.411; *p* = 0.019), and back stretch (r = 0.406; *p* = 0.021). Finally, to assess the potential predictive capacity of selected measures, we applied an exploratory logistic regression model with cross-validation.

Given the limited sample size (n = 32), the machine learning analyses were conducted in an exploratory manner to assess whether selected functional and physiological variables could provide preliminary classification of sleep quality. The logistic regression model using 5TSTS and TUG achieved a mean accuracy of 0.76 (±0.15), precision of 0.79 (±0.18), recall of 0.83 (±0.21), and ROC/AUC of 0.74 (±0.16) across 5-fold cross-validation. While the data showed a moderate association between functional lower-limb performance and sleep quality, the findings are tentative given the small and specific sample.

## 4. Discussion

This study aimed to understand the differences between sleep quality, physical fitness, body composition, cardiovascular measures, and their interplay with sleep quality. It was hypothesized that the variables of functional fitness, body composition, and cardiovascular measures explain sleep quality and its dependency. The results of the present study confirmed the hypothesis; however, while the findings revealed potential associations, they should be interpreted with caution, given the limited statistical power and small sample size. Different variables also explained the total sleep time.

### 4.1. Sleep Quality Comparisons

When comparing the participants between people with poor and good sleep quality, it is possible to find that the 5TSTS, Back Stretch, and total sleep time significantly differed between groups, where the people with good sleep quality presented higher scores of functional fitness. These exploratory results suggest a positive association between physical fitness levels, particularly in lower body strength and flexibility, and sleep quality, which may contribute to improved sleep patterns. The literature provides supportive information about the relationship between functional fitness and sleep quality. Studies have shown that improvements in functional fitness, including lower limb strength, have been associated with improved sleep outcomes [47,49,50]. Interventions that target lower limb strength, such as resistance training and specific exercises like yoga, have been proven to enhance sleep quality and overall wellbeing [51,52,53]. Lower limb strength plays an important role in mobility, and independence is highlighted as an important factor in supporting a healthy sleep pattern [47,54,55].

### 4.2. Associative Analysis and Machine Learning

The associative analysis allowed us to highlight the variables that primarily explain the total sleep time. For the total sleep, the Back Stretch, waist girth, 5TSTS, and Visceral Fat explained the sleep time. The sleep quality relationships with waist girth, visceral fat, and total fat can be explained by the body composition interplay with hormonal regulation, inflammation, and metabolic health [56,57]. Additionally, variables like 5TSTS, Back Stretch, and Seat and Reach test reflect aspects of functional fitness and flexibility, and again may influence comfort and relaxation [56,58,59].

The negative impact of body water is possibly explained by the increased urine output, resulting in the need for frequent urination during the night [60,61,62]. This negative effect on the circadian rhythm due to urine production can interfere with sleep continuity, resulting in poor sleep quality [62,63]. As for the 2MW, the literature presents evidence of the positive effects of aerobic exercise on arterial pressure and cardiovascular health [64,65]. Lately, aerobic exercise seems related to good sleep quality, time, efficiency, and latency [66,67,68].

The logistic regression model using 5TSTS and TUG achieved a mean accuracy of 0.76, precision of 0.79, recall of 0.83, and ROC/AUC of 0.74. These results suggest that functional fitness variables, particularly strength-related metrics, may serve as possible meaningful predictors of poor versus good sleep quality in older adults. This is aligned with the literature where body composition [56,57] and functional fitness [56,58,59] seem to be associated with good sleep quality and wellbeing. However, it was not possible to find studies with machine learning analysis to predict sleep quality based on anthropometrics, body composition, and functional fitness. For those reasons, comparisons with other studies regarding the algorithm’s scores were difficult to perform. Anyway, other studies with accelerometers and electrodermal instruments reported that machine learning algorithms may predict sleep quality with a percentage of accuracy between 78% and 84% [69,70], aligned with the present study. The use of a limited number of predictors in the machine learning models allowed for greater interpretability and reduced the risk of overfitting given the small sample size [71]. However, this parsimony may overlook important variables and complex interactions, limiting predictive accuracy. Thus, the findings are preliminary and hypothesis-generating, requiring validation in larger, more diverse samples with broader variable sets.

### 4.3. Psychophysiological Remarks

Considering a psychophysiological approach, the interplay between lower limb strength and sleep quality can be explained by different mechanisms. Lower limb strength and consequent independence may promote physical activity and tiredness, resulting in relaxation and somnolence [72,73]. Second, the lower limbs’ strength, relation to balance and stability may contribute to a perception of safety due to the reduced risk of falls and injuries during sleep, contributing to better sleep quality [72,74] because lower limb-related physical fitness is associated with reduced levels of pain and discomfort that may disrupt sleep [73]. Altogether, psychologically, these implications may also result in positive mental health and relaxation, resulting in better sleep quality [47,75]. Finally, physical activity and exercise regulate circadian rhythms and release endorphins, which are known to enhance sleep patterns [52,76].

### 4.4. Strengths, Limitations and Future Studies

This study presents a novel and exploratory approach to understanding the relationship between body composition, functional fitness, and sleep quality in older adults. A key strength lies in the integration of traditional statistical methods with a machine learning algorithm (logistic regression), which provides complementary perspectives on the data and enhances analytical robustness. The study uses validated tools such as the Pittsburgh Sleep Quality Index and BIA-based body composition estimates, alongside functional fitness tests, offering a multidimensional profile of the participants. Furthermore, the use of cross-validation and transparent model evaluation metrics (e.g., ROC AUC, accuracy, precision, recall) strengthens the internal consistency of the machine learning analysis and reflects a commitment to methodological rigor despite the exploratory nature of the work.

It is also possible to point out some of the limitations of this study: (i) this is not an interventional study comparing exercise base therapeutics; (ii) this study did not control biochemical, psychological, or physiological variables; (iii) the daily life routines including physical activity and nutritional habits were not evaluated or controlled; (iv) the sample size and the number of males and the exploratory characteristics of the study did not allow us to conduct sex comparisons with precise outputs. Although logistic regression modeling was performed with only two predictors to avoid overfitting, still limited the power and generalizability of our results, additionally no factor analysis were made to select important variables; (v) the reliance on estimated data from bioimpedance and subjective sleep measures (PSQI) introduces potential measurement bias, additionally, body composition estimates were derived from BIA using proprietary algorithms, which may lack validation across diverse populations, reducing reproducibility compared to reference methods such as DEXA; (vi) the machine learning algorithms were technically valid for application in this dataset. Further, future studies should (i) evaluate the effects of a regular training program on sleep quality; (ii) analyze biochemical, psychological, and physiological variables as well as body composition and physical fitness; (iii) assess the effects of active living and wellbeing lifestyles in sleep quality; (iiii) performing more robust approaches, such as classificatory machine learning approach, that can deal with several characteristics of predictors in the same set of analysis; (iv) recruit larger samples; (iv) mitigate the lack of data collection about bruxism incidence in the participants, which has been reported as a significant co-factor in sleep worsening; (v) employ precise instruments like DEXA and accelerometers to evaluate body composition and sleep quality.

## 5. Conclusions

This exploratory analysis provides early insight into potential functional predictors of sleep quality, which may inform future confirmatory research with larger samples that physical fitness and body composition may be important in sleep quality. The lower limbs’ strength and upper limbs’ flexibility seem to explain the sleep quality. This study suggests that improving muscle strength and managing body fat levels through regular physical activity may contribute to better sleep quality in older adults. Integrating simple functional fitness assessments (such as the 5TSTS) into routine geriatric evaluations may provide a practical pathway to identify older adults at higher risk of poor sleep quality and associated health outcomes.

## Figures and Tables

**Table 1:** Comparisons between sleep quality.

Variables	Good Sleep Quality (n = 15)	Poor Sleep Quality (n = 17)	Sleep Quality Comparison		
	Mean (±SD)	Mean (±SD)	t	*p*	η^2^
Age (yo)	69.47 (±5.99)	73.24 (±7.34)	−1.724	0.095	0.456
Mass (kg)	64.73 (±11.87)	67.44 (±9.25)	−0.724	0.474	0.002
Stature (cm)	157.49 (±4.30)	159.16 (±5.58)	−0.938	0.356	0.013
Rest Heart rate (Bpm)	71.82 (±6.26)	72.38 (±8.91)	1.742	0.197	0.411
Hand grip (kg)	21.67 (±8.61)	26.12 (±5.46)	0.411	0.526	0.002
Arm curl (Repetition)	21.29 (±4.75)	23.30 (±4.00)	−0.204	0.840	0.408
Waist circumference (cm)	88.00 (±10.23)	89.05 (±9.34)	−1.766	0.088	0.178
Hip circumference (cm)	101.53 (±9.68)	99.62 (±4.86)	−1.302	0.203	0.634
5TSTS (seconds)	7.69 (±1.00)	6.75 (±1.08)	−0.303	0.764	0.164
CS30 (repetitions)	21.14 (±4.05)	22.84 (±3.30)	0.691	0.497	0.138
TUG (seconds)	5.96 (±1.04)	5.36 (±0.67)	2.564	0.016 *	0.476
Seat and Reach (cm)	−2.14 (±7.39)	−0.15 (±9.92)	−1.309	0.200	0.733
Back Stretch (cm)	−5.21 (±8.40)	−14.49 (±11.40)	1.957	0.060	0.188
2MST (Repetitions)	180.52 (±22.38)	186.69 (±35.45)	−0.637	0.529	0.453
Total Fat (kg)	20.94 (±7.33)	20.05 (±5.27)	2.592	0.015 *	0.239
Total Fat (%)	32.13 (±6.79)	29.93 (±5.78)	−0.580	0.566	0.148
Lean Mass (kg)	41.23 (±5.60)	44.54 (±6.37)	0.990	0.330	0.337
Lean Mass (%)	63.73 (±7.54)	64.88 (±7.49)	−1.552	0.131	0.157
Body Water (%)	47.95 (±4.70)	49.33 (±3.83)	−0.430	0.670	0.431
Visceral Fat	7.47 (±2.23)	8.29 (±3.42)	−0.911	0.369	0.344
MET [KJ]	5405.20 (±709.26)	5648.75 (±599.06)	−0.792	0.435	0.000
MET [Kcal]	1411.87 (±521.07)	1477.87 (±463.82)	−0.379	0.707	0.438
Total Sleep	6.13 (±1.46)	3.35 (±0.61)	6.882	<0.001 *	0.675

Note: yo: years old; Bpm: beats per minute; 5TSTS: Five Times Sit to Stand Test; CS30: Sit to stand test 30 s; TUG: time up and go-test; 2MST: 2 min step test; Kcal: kilocalories; * *p* < 0.05.

## Data Availability

The data presented in this study are available on request from the corresponding author due to possible participants identification.
